# Supplementary material for: Comprehensive Ecotoxicity Studies on Quaternary Ammonium Salts Synthesized from Vitamin B3 Supported by QSAR Calculations
Source: Plants (Basel). 2023 Feb 17;12(4):914. doi: 10.3390/plants12040914 (PMC9960687; doi:10.3390/plants12040914)
Supplement: Supplementary file 1 [file plants-12-00914-s001.zip › plants-2196634-supplementary.pdf]

ELECTRONIC SUPPLEMENTARY INFORMATION

Comprehensive Ecotoxicity Studies on Quaternary Ammonium Salts Synthesized from Vitamin B_3 Supported by QSAR Calculations

Aleksandra Nowacka, Adriana Olejniczak, Witold Stachowiak, Michał Niemczak*

Faculty of Chemical Technology, Poznan University of Technology, 60-965 Poznan, Poland

** Correspondence: michal.niemczak@put.poznan.pl*

Table S1. Mean stem and root lengths and calculated SEM for white mustard.

Compound	Concentration [mg/kg]	1st repetition Mean value [cm]		1st repetition SEM [%]		2nd repetition Mean value [cm]		2nd repetition SEM [%]	
		Root	Stem	Root	Stem	Root	Stem	Root	Stem
**Control**	0	5.71	12.31	13	6	4.62	10.09	10	8
**NA**	10	6.57	11.13	12	9	2.53	9.46	5	3
**NA**	100	5.68	11.61	11	2	2.75	9.56	5	8
**NA**	1000	6.74	13.29	11	4	3.81	11.07	8	5
**[C_4NA][Br]**	10	5.31	11.27	13	11	5.01	10.54	10	7
**[C_4NA][Br]**	100	4.78	11.78	7	3	3.82	9.94	10	4
**[C_4NA][Br]**	1000	3.65	10.84	6	3	3.75	11.09	6	8
**[C_{10}NA][Br]**	10	6.16	12.63	12	4	4.03	9.83	10	10
**[C_{10}NA][Br]**	100	6.05	12.60	9	4	3.18	8.83	9	8
**[C_{10}NA][Br]**	1000	6.64	11.87	8	3	4.01	10.61	8	4
**[C_{16}NA][Br]**	10	6.24	12.03	11	5	3.21	8.81	7	5
**[C_{16}NA][Br]**	100	7.50	12.20	5	5	3.50	9.52	9	5
**[C_{16}NA][Br]**	1000	7.64	13.3	6	3	2.69	8.84	5	7

H (Test statistic): 20.69 for roots, 15.41 for stems; p (probability value): 0.055 for roots, 0.22 for stems

Table S2. Mean stem and root lengths and calculated SEM for sorghum.

Compound	Concentration [mg/kg]	Mean value [cm]		SEM [%]	
		Root	Stem	Root	Stem
**Control**	0	8.21	12.25	10	3
**NA**	10	9.17	9.92	17	4
**NA**	100	7.98	10.05	7	6
**NA**	1000	8.03	10.58	14	9
**[C_4NA][Br]**	10	9.55	9.79	16	9
**[C_4NA][Br]**	100	7.04	8.49	13	12
**[C_4NA][Br]**	1000	9.57	9.95	8	9
**[C_{10}NA][Br]**	10	9.39	10.89	13	7
**[C_{10}NA][Br]**	100	7.75	11.80	16	12
**[C_{10}NA][Br]**	1000	9.46	11.03	8	5
**[C_{16}NA][Br]**	10	8.36	10.37	7	3
**[C_{16}NA][Br]**	100	8.59	8.72	12	12
**[C_{16}NA][Br]**	1000	7.74	9.52	15	10

H (Test statistic): 11.51 for roots, 13.167 for stems; p (probability value): 0.486 for roots, 0.357 for stems

HAZARDS TO THE AQUATIC ENVIRONMENT

Initial note regarding toxicity mechanisms: Neutral organics mainly present toxicity to organisms *via* narcosis, which is referred to baseline toxicity. Some types of organic chemicals demonstrate a more specific mode of toxicity based on the presence of reactive functional groups. The toxic effects of electrophiles (including amides) are based on their reaction with nucleophilic sites in biological macro molecules, but they cannot be defined in terms of a single mechanism of action. However, LC_{50} concentrations of directly acting electrophiles are generally lower than those of unreactive organic chemicals [60]. According to many reports, the toxicity of surfactants (as compounds exhibiting amphiphilic properties) is mainly caused by disruption of the cell membrane [41].

The Global Harmonized System (GHS) [26] consists of three short-term (acute) classification categories and four long-term (chronic) classification categories. The acute and chronic classification categories are applied independently. Criteria for classification of a substance into acute categories I to III are defined based only on acute toxicity data only (EC_{50} or LC_{50}). The criteria for classification of a substance into chronic categories combine two types of information, *i.e.* acute toxicity data and environmental fate data (degradability and bioaccumulation data). To classify a substance as 'hazardous to the aquatic environment' appropriate criteria must be met. First, toxicity to three representative aquatic species must be assessed (96h Fish, 48h crustacea, 72 or 96h for algae or other aquatic plant such as *L. minor*). However, to be classified into chronic category, the substance must be not rapidly degradable and simultaneously its log K_{OW} must be equal or greater than 4 (if BCF is not available).

Table S3. Short-term toxicity assessment.

GHS category	Concentration [mg/L][a]	Acute toxicity
**None**	Above 100	---
**Acute III**	10-100	Harmful to aquatic life
**Acute II**	1-10	Toxic to aquatic life
**Acute I**	<1	Very toxic to aquatic life

a: EC_{50} or LC_{50}

Table S4. Long-term toxicity assessment for substances for which adequate chronic toxicity data are not available.

GHS category	Concentration [mg/L][a]	Chronic toxicity
**None**	Above 100	---
**Chronic III**	10-100	Harmful to aquatic life with long lasting effects
**Chronic II**	1-10	Toxic to aquatic life with long lasting effects
**Chronic I**	<1	Very toxic to aquatic life with long lasting effects

a: EC_{50} or LC_{50}

Note: Additionally, there is the Chronic IV category '*May cause long lasting harmful effects on aquatic life*' for poorly soluble substances, for which no acute toxicity was recorded at levels up to the water solubility. If they simultaneously are not rapidly degradable and have a log K_{OW} ≥ 4, indicating a potential to bioaccumulation, such compounds should be classified into this category (unless there is other scientific evidence that clearly demonstrates that this classification is unnecessary).

Table S5. The simplified Molecular Input Line Entry System (SMILES) used to input chemical structures into computer software.

Compound	SMILES
**NA**	NC(=O)c1cccnc1
**[C_4NA][Br]**	[Br-].CCCC[n+]1cccc(c1)C(N)=O
**[C_{10}NA][Br]**	[Br-].CCCCCCCCCC[n+]1cccc(c1)C(N)=O
**[C_{16}NA][Br]**	[Br-].CCCCCCCCCCCCCCCC[n+]1cccc(c1)C(N)=O

Table S6. The predicted toxicity for compounds assessed as neutral organics.

			NA		$[C_4NA][Br]$		$[C_{10}NA][Br]$		$[C_{16}NA][Br]$		
		**Log K_{OW}**[a]	-0.45	-0.66	-3.10	-1.41	-0.15	-0.18	2.80	1.70	
**Organism**	**Duration**	**Endpoint**	**Concentration [mg/L]**								**Max Log K_{OW}**
Fish	96 hours	LC_{50}	16000	24000	NES[b]	260000	24000	6800	68	NES	5
Daphnid	48 hours	LC_{50}	7200	11000	2900000	110000	11000	3400	41	NES	5
Green Algae	96 hours	EC_{50}	2100	2900	300000	21000	3700	1400	42	190	6.4
Fish		ChV[c]	1200	1700	450000	17000	1900	560	7.2	45	8
Daphnid		ChV	380	520	75000	4300	630	220	5.0	25	8
Green Algae		ChV	330	430	28000	2800	620	270	13	47	8
Fish (SW)[d]	96 hours	LC_{50}	20000	29000	NES	320000	30000	8500	86	NES	5
Mysid	96 hours	LC_{50}	77000	130000	NES	2400000	96000	18000	37	NES	5
Fish (SW)		ChV	480	630	47000	4200	870	360	15	59	8
Mysid (SW)		ChV	14000	25000	NES	570000	16000	2400	2.5	43	8
Earthworm	14 days	LC_{50}	380	400	1500	1000	1000	860	NES	NES	6

The results have been rounded to two significant digits. a: For each compound, a prediction was made for two log K_{OW}, predicted and measured, respectively; b: The underlining means that concentration is above the solubility of chemical - if it is 10 times or even greater, there is no effect at saturation (NES); c: To calculate a NOEC, the Chronic Value (ChV) can be divided by √2 (REACH Guidance R10, Table R10-1); d: salt water

Table S7. The predicted toxicity for compounds assessed as amides.

			NA		$[C_4NA][Br]$		$[C_{10}NA][Br]$		$[C_{16}NA][Br]$		
		**Log K_{OW}[a]**	-0.45	-0.66	-3.10	-1.41	-0.15	-0.18	2.80	1.70	
**Organism**	**Duration**	**Endpoint**	**Concentration [mg/L]**								**Max Log K_{OW}**
Fish	96 hours	LC_{50}	2800	3900	69000	34000	4600	1500	29	150	5
Daphnid	48 hours	LC_{50}	3900	5700	1400000[b]	56000	6200	1900	25	150	5
Green Algae	96 hours	EC_{50}	140	180	13000	1200	250	100	4.6	18	6.4
Fish		ChV[c]	12	16	960	100	23	10	0.53	1.9	8
Daphnid		ChV	260	360	37000	2600	460	180	5.5	24	8
Green Algae		ChV	30	36	880	170	63	34	4.2	11	8
Fish (SW)[d]	96 hours	LC_{50}	1800	2400	290000	19000	3100	1100	31	140	5
Mysid (SW)	96 hours	LC_{50}	52	67	3600	400	98	44	2.5	8.6	5
Mysid (SW)		ChV	52	79	39000	960	75	19	0.14	1.1	8
Earthworm	14 days	LC_{50}	880	940	4900	2700	2200	1800	NES	NES	6

The results have been rounded to two significant digits. a: For each compound, a prediction was made for two log K_{OW}, predicted and measured, respectively; b: The underlining means that concentration is above the solubility of chemical - if it is 10 times or even greater, there is no effect at saturation (NES); c: To calculate a NOEC, the Chronic Value (ChV) can be divided by √2 (REACH Guidance R10, Table R10-1); d: salt water

Note: The K_{OW} is the main physicochemical attribute directly correlates the chemical structure with a toxic effect for non-reactive neutral organic chemicals. Although in the case of cationic surfactants, the lipophilic alkyl chain length is used as a descriptor. This is due to the lack of a validated method for determining the octanol-water partition coefficient in their case - this particular issue was extensively studied and described by Hodges et al. [61].

Table S8. The predicted toxicity for surface-active compounds, assessed as special class cationic surfactants.

		$[C_{10}NA][Br]$	$[C_{16}NA][Br]$
**Organism**	**Duration**	**Concentration [mg/L]**	
Fish	96 hours	54	1.90
Daphnid	48 hours	5.9	1.60
Snail	96 hours	No data	0.68

The results have been rounded to two significant digits. Endpoints were LC_{50}.

Final conclusions regarding potential hazards to the aquatic environment

1. *Acute toxicity*: Traditionally, the most conservative effect level of the multiple estimates for a given endpoint is selected when the predictions are identified from multiple classes. In that case, the best approach is compatible with traditional, because on the basis of the structure and mechanism of action, **NA** and **$[C_4NA][Br]$** should be assessed as amide class. **$[C_{10}NA]$ $[Br]$** and **$[C_{16}NA][Br]$** should be evaluated as cationic surfactants, because their toxicity will be mainly caused by the disruption of the cell membrane [41]. In these cases there is no appropriate equation to predict toxicity to water plants, however, practical tests performed by us on *L. minor* revealed that EC_{50} after 72h for **$[C_{10}NA][Br]$** was within a range of 100-1000 mg/L, while for **$[C_{16}NA][Br]$** was within 10-24 mg/L. Gathered data allow to conclude, that these two compounds should be categorized in acute II category, which means that they exhibit toxicity to aquatic life. Although their much lower experimental toxicity to aquatic plants in comparison to predicted toxicity to fish and daphnids points out the plausible hypothesis that some of these results can be underestimated. The QSAR models for predicting toxicity of cationic surfactants were built on fully synthetic, simple compounds and can be less precise in the case of naturally based surfactants that can be less toxic than their fully synthetic analogs. There is a possibility that experiments performed in the near future will allow to reassess these compounds and provide more accurate QSAR models.

2. *Bioaccumulation*: All compounds possess log K_{OW} lower than 4 (predicted and as well as measured experimentally). Additionally, all of them are characterized by BCF (Bioconcentration Factor) smaller than 500 (log BCF < 2.7, log BCF for **$[C_{16}NA][Br]$** was estimated as 1.85, for other compounds it was equal to 0.5), which indicates that they do not exhibit potential for bioaccumulation in organisms at various trophic levels. According to BIOWIN models 1, 2 and 4, all compounds should biodegrade fast, however, due to results obtained from models number 3, 5, 6 and 7, compounds should be assessed as not readily biodegradable. The results from activated sludge models were summarized in Table 4 (main article). Therefore, QSAR models are not yet sufficiently accurate to predict ready biodegradation, while reliable toxicity and log K_{OW} data, obtained by using QSAR, allow for the environmental risk assessment [26].

Final remark: On the basis of the mentioned-above deductions, **NA** and **$[C_4NA][Br]$** should not pose a threat to aquatic life, however, **$[C_{10}NA][Br]$** and **$[C_{16}NA][Br]$** should be classified in acute II aquatic toxicity (toxic to aquatic life).

Table S9. Toxicity towards *L. minor* based on the number of fronds after 72 hours (3 days).

	Concentration [mg/L]									
	0.1		1		10		100		1000	
**Compound**	**GI[a] [%]**	**SEM[b] [%]**	**GI [%]**	**SEM [%]**	**GI [%]**	**SEM [%]**	**GI [%]**	**SEM [%]**	**GI [%]**	**SEM [%]**
**NA**	5	10	7	6	6	8	-3	9	16	6
**[C_4NA][Br]**	12	6	5	7	12	9	8	3	42	6
**[C_{10}NA][Br]**	5	8	-12	17	-8	11	37	8	100	0
**[C_{16}NA][Br]**	-14	16	6	21	30	21	---[c]	---	---	---

a: growth inhibition; b: standard error; c: the highest possible concentration for this compound was equal to 24 mg/L and resulted in a growth reduction of 67% (SEM = 8%)

Table S10. Toxicity towards *L. minor* based on the number of fronds after 7 days.

	Concentration [mg/L]									
	0.1		1		10		100		1000	
**Compound**	**GI[a] [%]**	**SEM[b] [%]**	**GI [%]**	**SEM [%]**	**GI [%]**	**SEM [%]**	**GI [%]**	**SEM [%]**	**GI [%]**	**SEM [%]**
**NA**	0	12	8	3	8	3	0	13	14	5
**[C_4NA][Br]**	6	4	-9	2	-5	15	-8	5	48	4
**[C_{10}NA][Br]**	6	5	-2	6	-4	5	45	9	100	0
**[C_{16}NA][Br]**	-12	10	6	17	47	9	---[c]	---	---	---

a: growth inhibition; b: standard error; c: the highest possible concentration for this compound was equal to 24 mg/L and resulted in a growth reduction of 68% (SEM = 9%)

References

26. United Nations. Globally Harmonized System of Classification and Labelling of Chemicals (GHS); United Nations: New York, NY, USA, 2021.

41. Kumari, P.; Pillai, V.V.S.; Benedetto, A. Mechanisms of Action of Ionic Liquids on Living Cells: The State of the Art. *Biophys. Rev.* **2020**, 12, 1187–1215, doi:10.1007/s12551-020-00754-w.

60. Hermens, J.L. Electrophiles and Acute Toxicity to Fish. *Environ. Health Perspect.* **1990**, 87, 219–225, doi:10.1289/ehp.9087219.

61. Hodges, G.; Eadsforth, C.; Bossuyt, B.; Bouvy, A.; Enrici, M.-H.; Geurts, M.; Kotthoff, M.; Michie, E.; Miller, D.; Müller, J.; et al. A Comparison of Log Kow (n-Octanol–Water Partition Coefficient) Values for Non-Ionic, Anionic, Cationic and Amphoteric Surfactants Determined Using Predictions and Experimental Methods. *Environ. Sci. Eur.* **2019**, 31, 1, doi:10.1186/s12302-018-0176-7.
